# Visual Osteoclast Fusion via A Fluorescence Method

**DOI:** 10.1038/s41598-018-28205-3

**Published:** 2018-07-05

**Authors:** Boer Li, Fanyuan Yu, Fanzi Wu, Ke Wang, Feng Lou, Demao Zhang, Xueyang Liao, Bei Yin, Chenglin Wang, Ling Ye

**Affiliations:** 1 State Key Laboratory of Oral Diseases, West China Hospital of Stomatology, Sichuan University, Chengdu, 610041 Sichuan China; 2 Department of Biomedical Sciences, Texas A&M College of Dentistry, Dallas, 75246 TX USA

## Abstract

Osteoclasts are multinucleated giant cells. Fusion is an essential element in the formation of osteoclasts. However, the exact cellular events and mechanisms remain largely unknown because of limited and insufficient methods for observing fusion process. In this work, a fluorescence reporter strategy was established to monitor osteoclast fusion. After fusing with cells expressing Cre recombinase, those cells with double fluorescence switch its expression from red to green fluorescent protein. The effect of RANKL and PTH on osteoclast fusion were both quantitatively and visually detected utilizing this strategy. Furthermore, a combination of this strategy with a technique of fluorescence-activated cell sorting revealed two different populations of fused osteoclasts, tdTomato+ GFP+ cells (TG cells) and GFP+ cells (G cells). The results argue for the potential of combining this technique with other bio-technologies to gain more information about osteoclast fusion. Overall, these data demonstrated that this visual fluorescence switch strategy is useful for further analysis of osteoclast fusion mechanisms.

## Introduction

Osteoclasts are a group of specialized cells that originate in hematopoietic precursors. They primarily derive from monocytes/macrophages and undergo differentiation followed by fusion to form multinucleated cells. Such mature osteoclasts are capable of forming bone-resorbing compartments^1^. They share similarities with monocyte/macrophages from precursors to preosteoclast^1,2^. However, fusion triggers off a process of intensified differentiation. Once fused polykaryon appears, it functions as the major bone destroyer^3^. After fusion, these multinucleated cells rapidly and substantially increase in size, thus extending the resorption area and intensifying the production of acids and digestive enzymes. This, in turn, empowers osteoclast efficient actions despite the short life span^4^. As expected, disorders of osteoclast fusion were reported to disrupt bone homeostasis. For instance, Paget’s disease entails an increase in the prevalence of multinucleated osteoclasts and a remarkable activation of bone resorption, which in turn leads to fragile bones with abnormal remodeling^5^. Moreover, knock out of DC-STAMP, OC-STAMP or ATP6V0d2 also significantly impairs osteoclast fusion and plays a role in the formation of rare multinucleated osteoclasts^6–8^. It is widely accepted that multi-nuclear osteoclasts are required to maintain bone balance, and researches have uncovered several important regulators of osteoclast fusion. The specific biological processes underlying fusion, however, are still poorly understood^9,10^.

In terms of methods, time-lapse microscopic imaging is a commonly used procedure in research of osteoclast fusion. But this method provides only limited information and often produces blurry images, thus creating constraints in research into these matters^4,9^. In 2004, Kondo *et al*. proposed retroviral vector packaging as a new detection method for the process of osteoclast fusion^10^. The co-culturing of packaging-deficient cells with packaging cells shows that retrovirus production can occur only as a result of cell-cell fusion. Their work offered another approach for the study of fusion. However, this method was not widely adopted because of the complexity of the processes involved and indirectness of the demonstration of cellular morphological changes during fusion^10^.

In 2010, yet another method was developed for observing fusion via the construction of an indicator plasmid with double fluorescence. Cells transfected with this indicator plasmid express a red fluorescence protein (RFP). This could then be deleted after fusing with Cre-transfected cells, paving the way for the subsequent translation of green fluorescence protein (GFP)^11^. At present, this strategy has not yet been used in studying osteoclast fusion. But it has successfully produced images whose quality is superior to that of the images produced by time-lapse microscopic techniques^11^. Inspired by this approach, the osteoclast fusion research presented here employed a new fluorescence switch strategy based on the Cre-loxP system. Visual osteoclast fusion was achieved through the co-culturing of bone marrow monocytes (BMMs) from *Cathepsin K-Cre (Ctsk-Cre)* transgenic mice and BMMs from *Rosa*^*mTmG*^ mice. *Rosa*^*mTmG*^ is a double-fluorescent Cre reporter mouse^12^. Upon the occurrence of Cre, a fluorescence switch located on the cell membrane is activated. In brief, if a Rosa^mTmG^ cell also contains Cre recombinase, it will express tdTomato (red) prior to excision and EGFP (green) after excision. *Ctsk-Cre* limits the expression of Cre to osteoclasts. Hence when fusion occurs between BMMs from *Ctsk-Cre* mice and those from *Rosa*^*mTmG*^ mice, EGFP positive (GFP+) cells will emerge. Such cells are fused cells. This technique provides a novel approach both for observing fusion and for detecting the factors responsible for the regulation of the fusion.

In order to validate the operational feasibility of this new approach, we carried out tests on several important regulators for osteoclastogenesis. The goal was to investigate whether these regulators also affect the fusion process. New insights gained via this research strategy validated the feasibility of this method. The research also provided several basic insights into the process of osteoclast fusion. This visual tool proved to have several advantages over the above-mentioned methods. In the first place, cell transfection is not required. This leads to largely simplified experimental procedures and reduced harm to cells. Besides, it can both provide real-time images and detect regulatory factors without the time-lapse microscopy or the virus packaging which are required in previous methods. Furthermore, the use of this method facilitates the sorting of fused osteoclasts via a combination of fluorescence-activated cell sorting (FACS) technique. This innovative approach also addresses the well-recognized need for greater knowledge of the precise mechanisms involved in osteoclast fusion. Its potential therapeutic value lies in the possible identification of those fusion events that can more specifically modulate bone resorption.

## Results

### Experimental design

**Figure 1:**
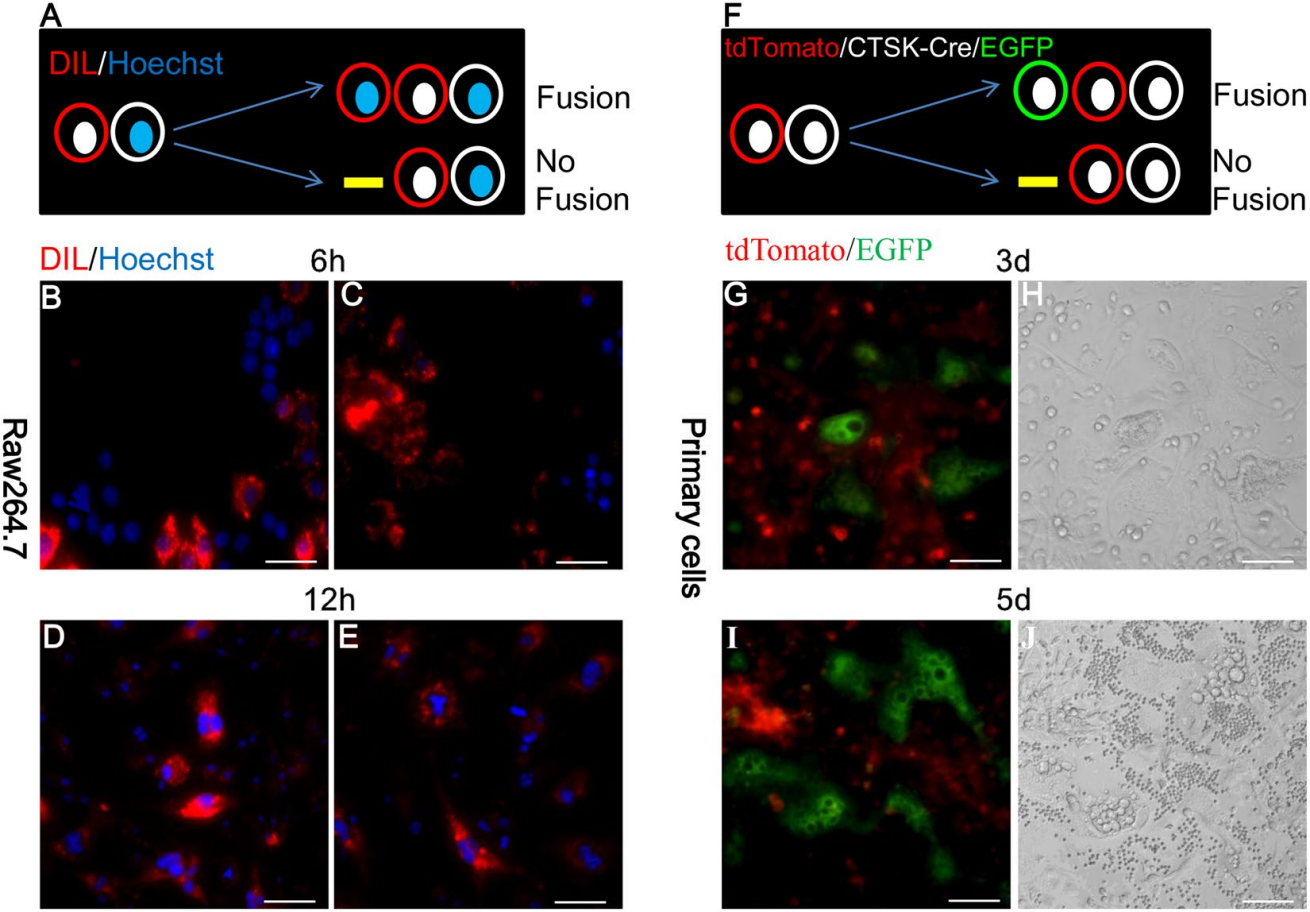
(**A**) Double fluorescence switch strategy for visualizing fusion. Preliminary tests of the fluorescence labelling method showed it to be a viable effective research tool. (**A**,**F**) A schematic presentation of the experiments. (**B**–**E)** Raw264.7 cells were treated with RANKL to produce fusion. (**G**–**J)** BMMs from *Ctsk-Cre* and *Rosa*^*mTmG*^ were 1:1 co-cultured and induced with an osteoclastogenic medium. Images from fluorescence microscope via this fluorescence switch strategy clearly and concisely presented fused GFP+ cells (**G**,**I**). In contrast to this strategy, the images produced by the traditional bright field method lose much information and make it difficult to specifically observe fused cells (H&J). All conclusions presented here reliably represent the results of experiments carried out at least three times. Scale bar: 50 um.

Preliminary tests were conducted to determine whether fluorescent labeling methods can be used to study osteoclast fusion. To begin, live Raw264.7 cells were divided into two groups; one was stained with DIL (red fluorescent cell membrane dye) and the other was stained with Hoechst (blue fluorescent nucleus dye). These two groups of cells were then co-cultured. If any fusion between them occurred, cells with the red fluorescent membrane and the blue fluorescent nucleus were observed. (A schematic presentation is shown in Fig. 1A,F). Six hours after RANKL stimulation, cells with red membrane and blue nucleus were observed. After 12 hours’ induction, these cells were found to have increased in size and to have acquired a larger number of nuclei (Fig. 1B–E). Subsequently, primary BMMs from *Ctsk-Cre* and *Rosa*^*mTmG*^ mice were co-cultured. As previously noted, GFP+ cells were subject to observation only if fusion had occurred (Fig. 1F). Three days after RANKL induction, a large number of GFP+ cells appeared and increased in size at day 5 (Fig. 1G,J). In comparison with bright field imaging techniques, this fluorescent switch method provides clearer images and substantially more information. Using this fluorescence switch strategy, an observer can easily detect fused cells and distinguish them from surrounding cells (Fig. 1G–J).

### *In Vivo* Imaging of *Ctsk-Cre; Rosa*^*mTmG*^ Mice

**Figure 2:**
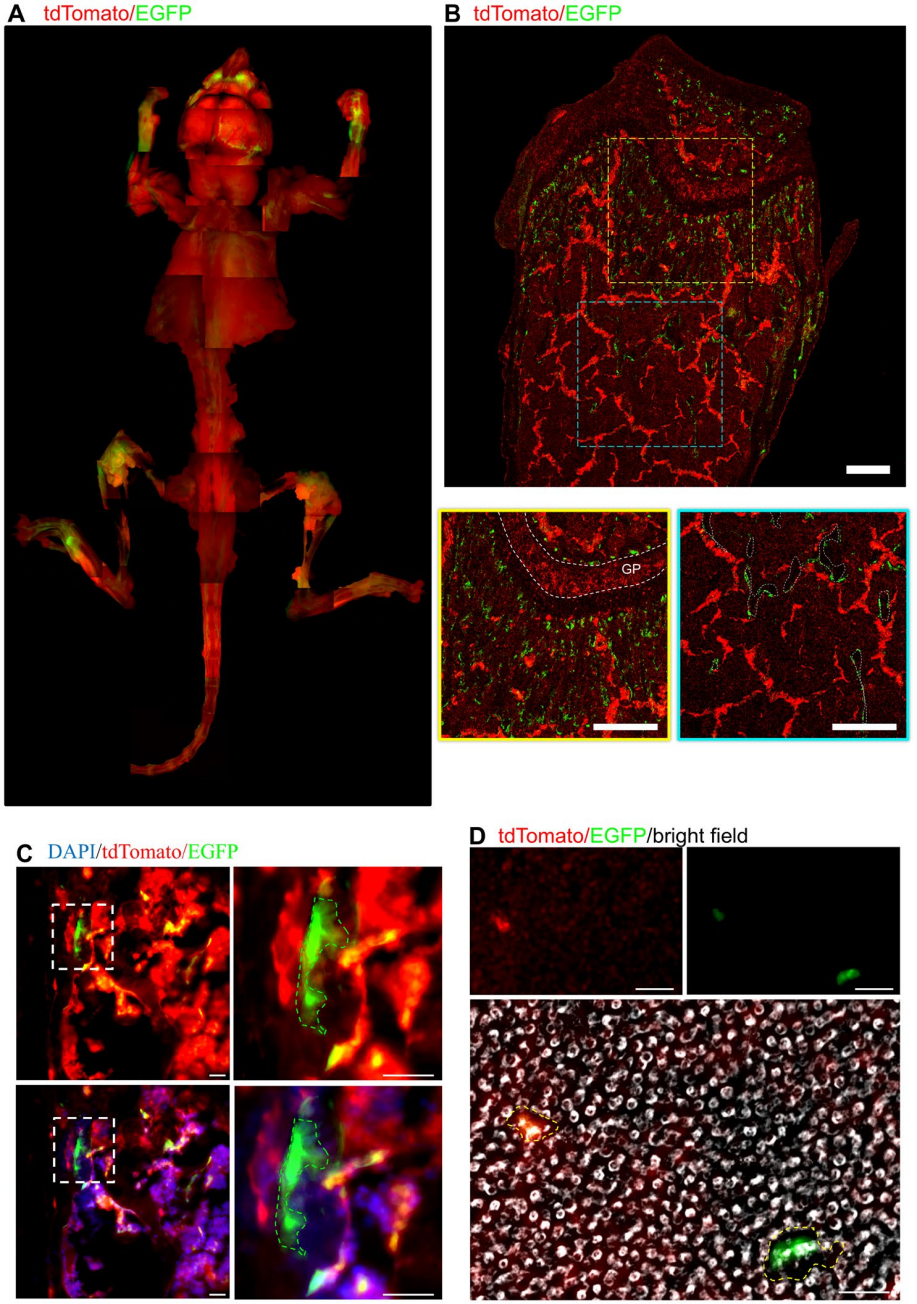
*In Vivo* imaging of *Ctsk-Cre; Rosa*^*mTmG*^ mice. (**A**) Stereo fluorescence microscopic images display the GFP positive cells distributed within the whole skeletal system of 2-month-old mice. (**B**) GFP positive cells are located principally on bone surfaces. Upper Panel: fluorescence image of the femur’s distal end. Lower Panels: Higher magnification of the yellow and blue dotted box regions, respectively. Yellow box: trabecular bone area beneath the growth plate (GP), the white dashed lines indicated the growth plate; Blue box: the middle region of the diaphysis, the white dashed lines indicated the trabecular bone. Scale bar: 100um. Additional information of this panel was clarified in Supplementary Figure S6. (**C**) High magnification images reveal that GFP positive cells increase substantially in size and take on a polarized shape as they adhere to bone surfaces. “Yellow” cells containing both EGFP and tdTomato were also detected. Scale bar: 50um. (**D**) A resorption analysis indicated that the resorption ability of GFP positive cells from *Ctsk-Cre;Rosa*^*mTmG*^ mice was much stronger than the resorption ability found in “yellow cells”. Scale bar: 100 µm.

Before further utilization of this fluorescent switch co-culture method, we identified and examined the fluorescent distributions in the skeletal system of *Ctsk-Cre Rosa*^*mTmG*^ mice *in vivo*. The stereo-fluorescence microscopic images clearly displayed the widespread presence of GFP+ cells in adult bones (Fig. 2A). Moreover, the femur sections demonstrated that the GFP+ cells were mainly located in trabecular bone surfaces (Figs 2B and S5, S6). More details emerged: high magnification imaging revealed the gigantic, multinucleated character of these GFP+ cells and their tendency to adhere to the bone surface. This distribution of GFP+ cells in bones was consistent with previous reports of osteoclast localization and also validated the use of Cre recombinase of this Cre line employed in this research^12,13^. Interestingly, in addition to these GFP+ cells, a population of “yellow cells” was found which were neither GFP+ cells nor simply red cells. They seemed to be of intermediate status, with both EGFP and tdTomato (Fig. 2C). Primary BMMs were isolated and cultured from *Ctsk-Cre;Rosa*^*mTmG*^ mice in collagen resorption analysis chambers. Six days after RANKL induction, obvious resorption lacuna appeared beneath GFP+ cells. Meanwhile, GFP mono-positive cells exhibited much stronger resorption results than yellow cells (Fig. 2D).

### Full-stage tracking of osteoclast fusion

**Figure 3:**
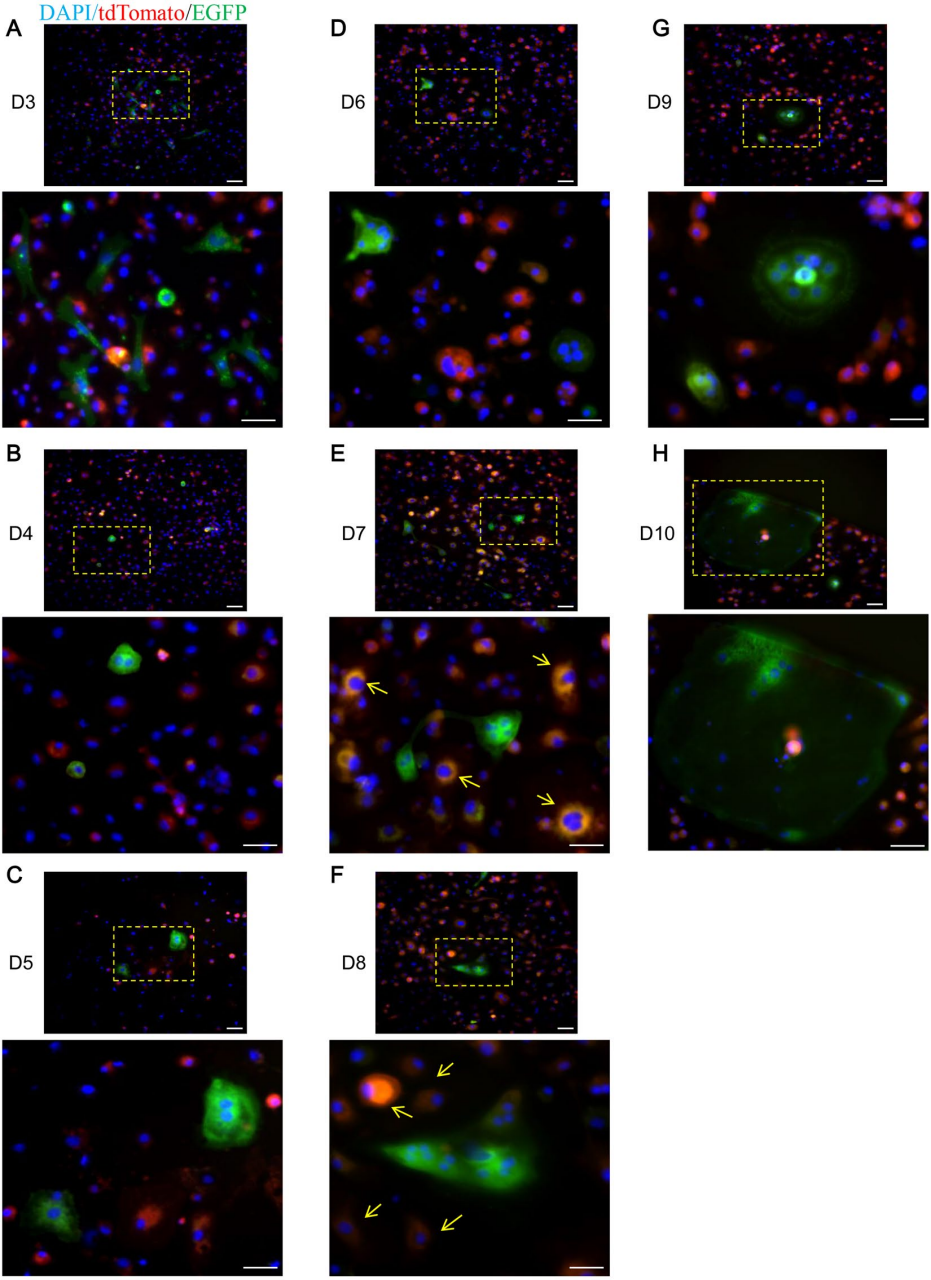
Full-stage tracking of osteoclast fusion. Co-cultured BMMs from *Ctsk-Cre* mice and *Rosa*^*mTmG*^ mice were induced with RANKL. GFP+ cells were observed on the third day, most of them containing only one single nucleus (**A**). Seven days after osteoclastogenic induction, fused multinucleated GFP+ cells were found in proximity to each other (**E**). From day3 to the final day of observation, GFP+ cells increased in size and became more polarized, with an increased number of nuclei (**A**–**H**). From day 7, nuclei (some of them condensed) were observed scattering with the cells. Also, on day 7 and day 8, the number of TG cells possessing both EGFP and tdTomato substantially increased and became easily observable (**E**–**H**). Scale bar: 50 um.

In order to further validate this new approach, a long-term tracking experiment was conducted to permit further observation of osteoclast fusion. Primary BMMs separated from *Ctsk-Cre* mice and *Rosa*^*mTmG*^ mice were co-cultured and treated with RANKL to initiate fusion. GFP+ cells appeared 3 days after RANKL induction. Notably, although these GFP+ cells had their origin in at least two cells (*Ctsk-Cre* BMM and *Rosa*^*mTmG*^ BMM), most of them had only a single nucleus at this stage (Fig. 3A). Later, the size of GFP+ cells increased, as did the number of nuclei. At the same time, the general morphology of GFP+ cells gradually changed, as their shape took on the greater spread and they became polarized (Fig. 3B–H). On day 7, many of the GFP+ cells moved close to each other, as an observable membrane approach occurred (Fig. 3E). This result was consistent with earlier research reports^9^. With respect to the nucleus, the number of nuclei in certain GFP+ cells increased along with the ongoing of differentiation. Besides, some of the nuclei were condensed and scattered, which is also consistent with previous research observations (Fig. 3E–H)^14^. Unexpectedly, from day3 to day10, a special population of “yellow cells” emerged (Fig. 3A–H). This was consistent with *in vivo* histological results from *Ctsk-Cre;Rosa*^*mTmG*^ mice (Fig. 2C).

### Major regulatory factors on osteoclast fusion

We conducted two experiments to test the potential utility of the fluorescence switch strategy for the identification of possible regulators of osteoclast fusion. Two important factors, RANKL and PTH, were tested for their possible effects on osteoclast fusion. At this point in time, the effect of regulatory cells on osteoclast fusion and the underlying mechanism were largely unknown. Therefore, this study introduced the technique to assess, for the first time, the possible influence of two major cell types, namely osteoblasts and osteocytes, on the process of osteoclast fusion.

#### RANKL and PTH

**Figure 4:**
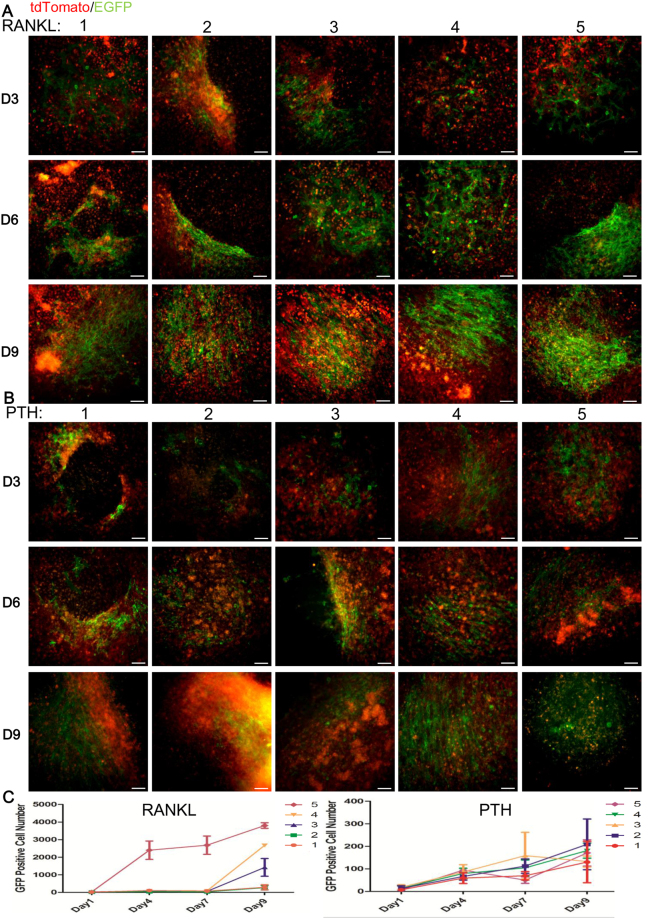
The functions of RANKL and PTH on osteoclast fusion. In the RANKL group, fused GFP+ cells increased along with the increased concentration of RANKL (**A**,**C)**. Concentrations of RANKL: 1, 10 ng/ml; 2, 25 ng/ml; 3, 50 ng/ml; 4, 75 ng/ml; 5, 100 ng/ml). PTH also induced fused GFP+ cells, but there was no correlation between the intensity and the concentration (**B**,**C**). Concentrations of PTH: 1, 10 ng/ml; 2, 25 ng/ml; 3, 50 ng/ml; 4, 75 ng/ml; 5, 100 ng/ml). Furthermore, when RANKL and PTH with the same level of concentration were used, they nonetheless differed in their ability to promote fusion. During the full range of observation, the RANKL showed stronger capacity to induce osteoclast fusion than the same concentration of PTH (**A**–**C**). Scale bar: 100 um. All error bars shown are the calculated standard deviation (SD) across triplicate experiments.

Although there is a long-standing debate about whether RANKL-independent osteoclastogenesis really occurs, RANKL is widely regarded as the most important required cytokine for osteoclast formation^1,15,16^. But the exact role of RANKL in osteoclast fusion remains unknown. We conducted an experiment to determine whether different concentrations of RANKL could influence osteoclast fusion in a dose-dependent manner. Our data indicated that RANKL does increase the number of GFP+ cells in a dose-dependent way from day 3 to the endpoint of observation (Fig. 4A), while BMM did not fuse and became GFP+ cells without RANKL induction (S7). Statistical analysis of GFP+ cells also gave quantitative confirmation that osteoclast fusion was indeed dependent on RANKL concentration. An increase in RANKL was found to enhance the process of fusion (Fig. 4C). Besides, it was confirmed that all GFP+ cells were TRAP+ by flow cytometry analysis (S8).

Another issue concerns the frequent use of PTH to reduce bone loss in contemporary clinical practice. But its use has resulted in dual effects on the skeletal system^17^. Intermittent usage can stimulate bone formation, whereas continuous secretion will lead to bone resorption^17^. Although previous reports showed that osteoclasts did not own PTH receptors, but this hormone affected osteoclastogenesis via influencing other bone cells, such as osteoblasts and osteocytes, there is as yet no evidence as to whether PTH exerts any impact on osteoclast fusion^18^. Our results documented that osteoclast fusion does occur in the presence of PTH (Fig. 4B). Because the primary culture of BMMs also contains other bone cells that have PTH receptors. But the ratio of fusion is dramatically lower than what occurs when RANKL induction is carried out at every observation point (Fig. 4). More importantly, the degree of fusion did not show a PTH concentration-dependent pattern, which is probably attributed to the indirect regulations of PTH for osteoclasts mentioned above (Fig. 4C).

#### Osteoblasts and Osteocytes

Recent studies have documented the regulatory functions of osteoblasts and osteocytes on osteoclastogenesis. No experimental evidence, however, is available on the question of whether these two types of cells affect the fusion process^19^. We decided to address this question. The following experiments were conducted. Osteoblasts or osteocytes were seeded as the bottom cells, whereas the top layer of cells consisted of BMMs co-cultured from *Ctsk-Cre* mice and *Rosa*^*mTmG*^ mice, as described in the methods section.

**Figure 5:**
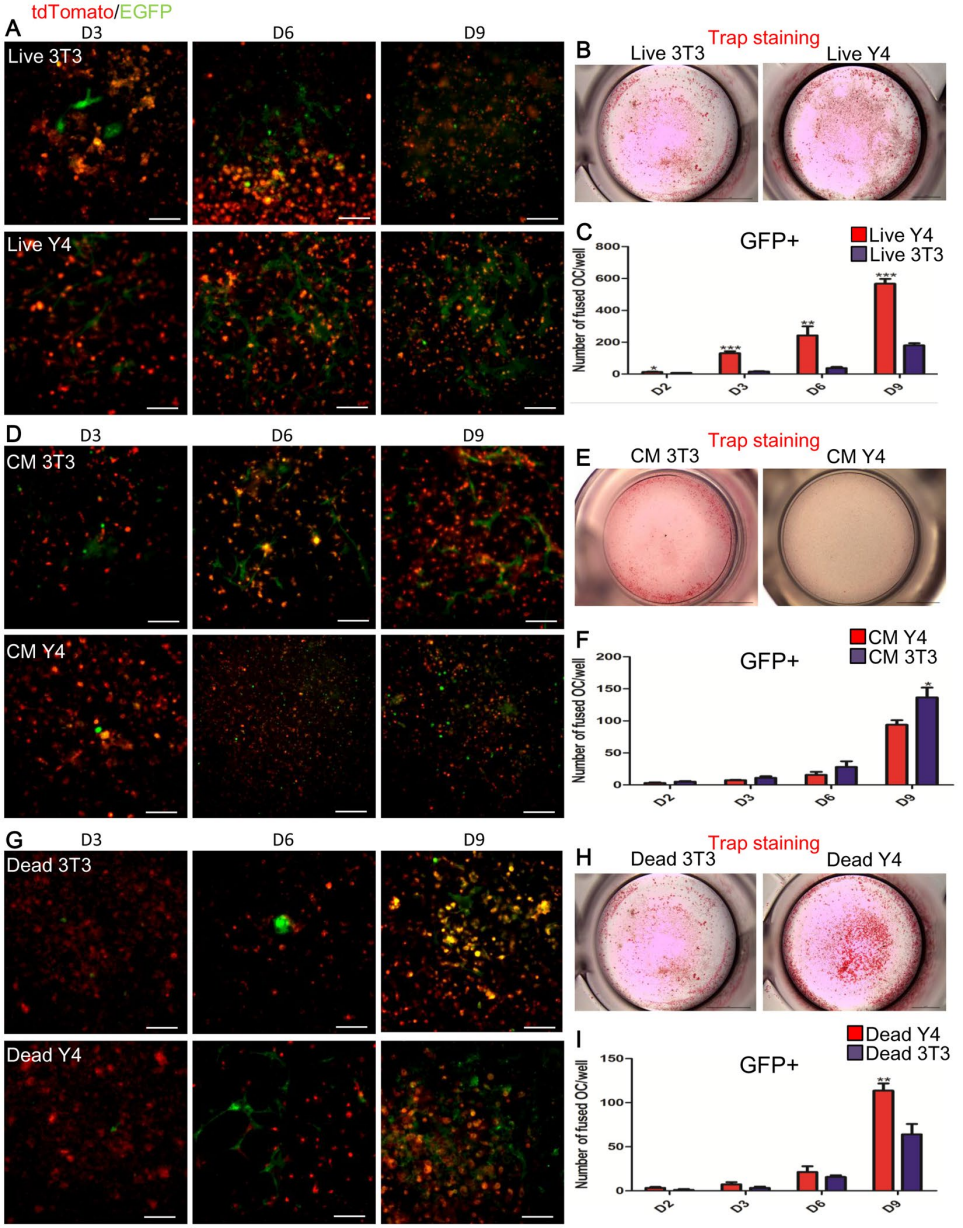
The functions of osteoblasts and osteocytes on osteoclast fusion. (**A**–**C)** In this “live” group, the bottom-level cells (all of them “live”) were directly co-cultured with top-level cells. (**D**–**F**) In this group (CM), the topmost cells were separated from the bottom cells by transwell chambers with a 0.4um pore size. This restricted communication of secreted matters. (**G**–**I**) In this “dead” group, bottom cells were rendered dead via application of 4%PFA. Under these conditions, only the membrane molecules of the bottom cells were able to interact with the top cells. The results indicate that osteocytes have much stronger ability than osteoblasts with respect to the promotion of osteoclast fusion (**A**–**C**). Secreted matters from osteoblasts were more effective in inducing fusion (**D**–**F**). However, osteocytes’ membrane-linked molecules produced significantly more fusion (**G**–**I**). Scare bar: 100um. 3T3: MC3T3-E1 cells as bottom cells; Y4: MLO-Y4 cells as bottom cells. All error bars shown are the calculated standard deviation (SD) across triplicate experiments. * for p < 0.05; **p < 0.01; ***p < 0.001.

In the first place, when the 1:1-mixed BMMs from *Ctsk-Cre* and *Rosa*^*mTmG*^ mice were separately cultured upon live MC3T3-E1 cells (osteoblastic-like cells) and live MLO-Y4 cells (osteocyte-like cells), the osteocytes exhibited a much stronger impact than the osteoblasts on the process of osteoclast fusion. More specifically, in the presence of live osteocytes, a significantly larger number of GFP+ cells were found than in the converse situation in which the bottom level consisted of osteoblasts (Fig. 5A–C). However, after the top cells were separated from the bottom cells by a transwell chamber, communication between the top and bottom cells was limited to secreted factors. In this situation, the secreted factors from bottom-level osteoblasts exerted a greater impact on osteoclast fusion than those derived from osteocytes (Fig. 5D–F).

Interestingly, in the “Dead” group, bottom cells were fixed in a manner that permitted interaction with top cells only through their membrane molecules. Dead osteocytes had a significantly stronger ability than dead osteoblasts to induce osteoclast fusion. Our results indicate that membrane-linked molecules from osteocytes have a significantly stronger capacity to induce osteoclast fusion than those from osteoblasts (Fig. 5G–I). Overall, osteocytes showed a stronger ability than osteoblasts to induce osteoclast fusion. And this capacity of osteocytes to induce fusion relies principally on membrane-linked molecules (Fig. 5).

### Two populations of fused osteoclasts

**Figure 6:**
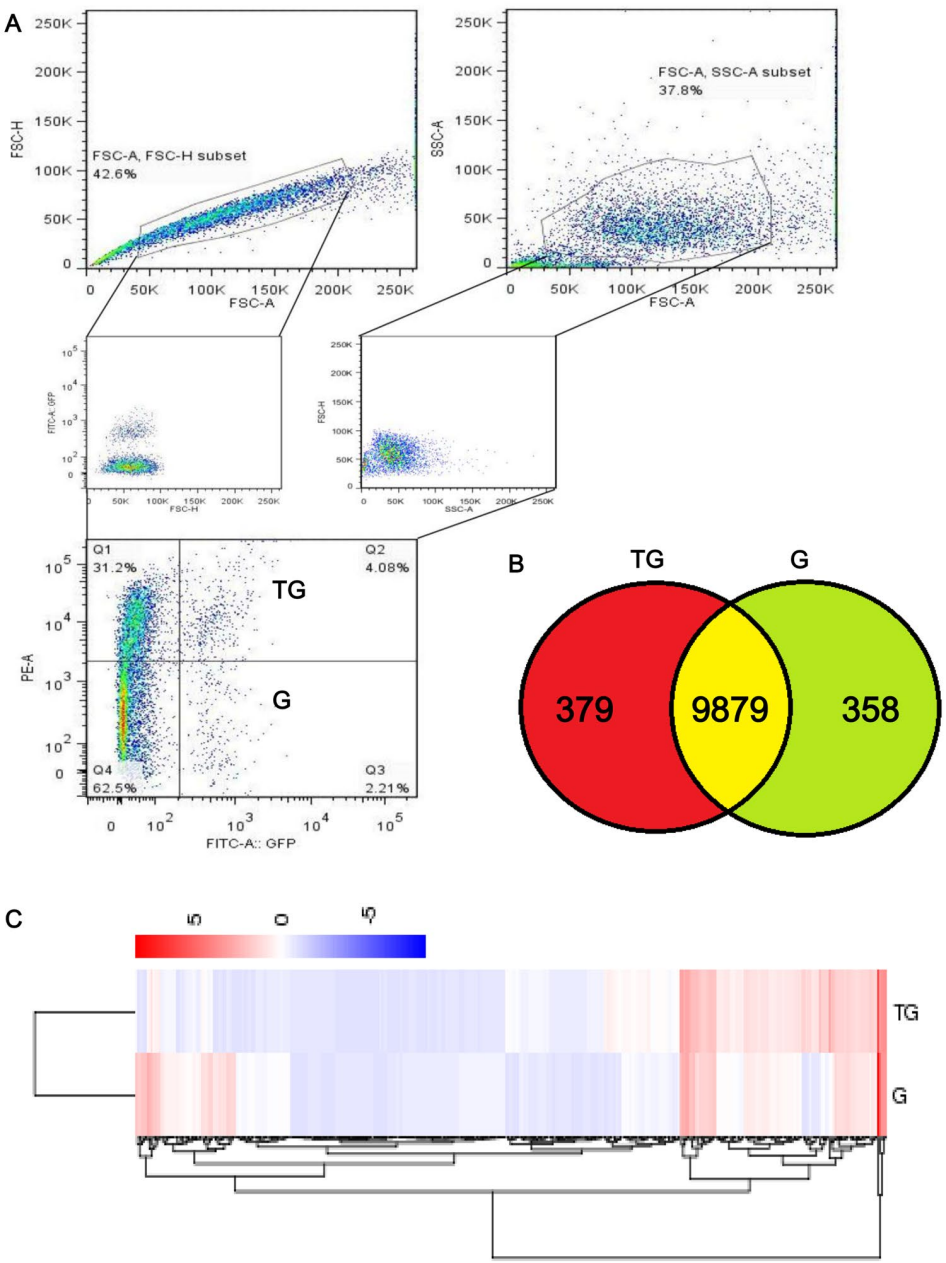
Fluorescence activated cell sorting (FACS) and high throughput mRNA-sequencing (RNA-Seq) for two observed populations of fused cells. Co-cultured BMMs from Ctsk-Cre mice and Rosa^mTmG^ mice were osteoclastogenically induced for 8 days. A FACS procedure was then used to separate and sort cells with mono-green fluorescence (G cells) and cells both with green and red fluorescence proteins (**A**). After being subjected to mRNA-seq (**C**), the TG cells and the G cells were found to have 9879 common genes. But 379 exclusive genes were expressed in the TG cells whereas 358 exclusive genes were expressed in the G cells (**B**).

From the results of *in vivo* procedures carried out on *Ctsk-Cre;Rosa*^*mTmG*^ mice, three types of cells, differentiated by color, were identified. In addition to mono-green (G cells) and mono-red (tdTomato+) cells, bone surfaces also harbored some possible intermediate cells which we have labeled “yellow cells”. They expressed both EGFP and tdTomato on membranes (abbreviated as TG cells, Figs 2 and S1–4). Moreover, long-term tracking images also revealed fluorescent heterogeneity *in vitro*. The concurrence of TG cells and G cells was clearly visible from day 6 (Fig. 3D–H). Further increasing the concentration of RANKL also altered the constitution of TG and G populations. In addition, the G/TG ratio rose with the up-regulation of RANKL (Fig. 4A). Subsequently, FACS was employed to isolate G and TG cells 8 days after RANKL induction (Fig. 6A). RNA-seq results indicated that TG and G expressed 9879 genes in common. But there were 379 genes’ exclusively expressed in TG cells and 358 genes exclusively expressed in G cells (Fig. 6B,C and Supplemental Table 1; detailed raw data of RNA-seq were listed in Supplemental Table 1). KEGG revealed that the exclusively expressed genes in TG cells and G cells had distinct cellular and molecular functions. For instance, the genes that are exclusively expressed in TG cells play a role in several processes, including cell cycle, apoptosis, carbohydrate digestion and absorption. They also play a role in the mTOR signaling pathway. Genes expressed by G cells, on the other hand, have potential roles in other processes: adhere junction, in starch and sucrose metabolism and in other glycan degradation respectively (S1). Further, GO enrichment analysis also indicated that the uniquely expressed genes in TG and G cells carried out different molecular functions (MF) and differed in terms of the generation of cellular components (CC) as well as in other biological processes (BP) (S2–4). These exclusively expressed genes in the two populations also displayed potential heterogeneity in fused cells, and they furthermore gave evidence of passing through two distinct phases during fusion.

## Discussion

Osteoclasts are essential to the maintenance of skeletal health because of the major (and perhaps not exclusive) role that they play in bone resorption^1,20^. Advances in osteoclast research can, therefore, have positive therapeutic implications by identifying potential interventions in the case of skeletal diseases. In this context, it can be considered a high research priority to shed more light on the precise mechanisms of osteoclastogenesis. Masaru *et al*. proposed that osteoclast fusion, as an essential element in the formation of osteoclasts, needs to be explored more carefully for purposes of drug design^4^. In view of our limited understanding of how this physiological process actually occurs, however, none of the currently available drugs was designed to target osteoclast fusion. The few methods currently available for studying osteoclast fusion suffer from several shortcomings, slowing down the progress of research on this topic. In this work, we have developed a Cre-based double fluorescence switch system to permit the actual real-time visual observation of cell fusion processes. The method also facilitates the detection of regulatory factors and the selection of fused cells. The use of this method, in short, has permitted us to observe the ongoing fusion process, hopefully opening up a methodological pathway to new insights into osteoclast biology.

Experiments were performed on the major regulators of osteoclast fusion. The results indicate that this tool could be useful in facilitating such analysis. Because the method involves simple procedures for observing the fusion process, it can potentially simplify research protocols and at the same time provide much more detailed information than previous methods. Quite apart from the qualitative morphological insights which the method generates, it can also facilitate quantitative analysis of the impact of regulators on osteoclast fusion. For example, by using a fluorescence microscope to count the numbers of GFP+ cells, we were able to detect differences between osteocytes and osteoblasts with respect to their differential impact on osteoclast fusion. The observations provided not only descriptive morphological insights but also quantifiable data as well. In addition to these studies on regulatory cells, however, we were also able to use the method to generate data on the differential impact of distinct concentrations of RANKL and PTH on osteoclast fusion. Our research suggests that the use of a florescence strategy provides insights, both scientifically reliable and clinically practical, on regulatory cytokines, hormones and other molecules involved in the fusion process.

More insights into the underlying mechanisms and cellular dynamics of osteoclast fusion are still required. We can anticipate that further research using this method will bring to light additional causal factors underlying the process of osteoclast fusion. For instance, if we were to combine, into one research protocol, the use of time-lapse fluorescence technology and the experimental modification of genes via Crisper-Cas9 or other gene editing technologies, we could anticipate even further insights, both morphological and statistical, into the genes involved in osteoclast fusion^21^.

Some issues remain with regard to the major regulators that were measured above. In the first place, though nuclear fusion and membrane fusion were both detected, the mechanisms have yet to be identified and require further research. How do osteoclasts move and migrate toward each other? How do they reorganize cytoskeletons during fusion? Answers to such questions would provide keys to uncovering the mechanisms of the membrane and nuclear fusion. Further research can use the method which we are proposing in combination with cytoskeletal fluorescence labeling and fluorescence time-lapse microscope to provide comprehensive insights into these processes and into the associated regulators. A point at issue is the emergence of the multiple nuclei that we detected in GFP+ cells. Although this phenomenon was discovered decades ago, we are still in the dark as to whether only one of the multiple nuclei is responsible for the transcription function or as to how osteoclasts select the nucleus that governs the fused cells^1,2^. Moreover, our research suggests that RANKL, a potential major regulating factor involved in osteoclast fusion, induce fusion via a concentration-dependent way. On the contrary, PTH showed a concentration independent pattern in osteoclast fusion process *in vitro* because osteoclasts do not own PTH receptors. Hence the regulation of PTH for osteoclasts fusion was mediated by the presence of other cells that have PTH receptors in the primary culture of BMMs. But precisely how RANKL achieves its impact on fusion is a matter for further research.

The data from our research has provided some new insights regarding the major regulatory cells involved in osteoclast fusion. It is known that the formation of multinucleated osteoclasts requires coordination between preosteoclasts and other cells. In the past, lining cells and osteoblasts were proposed as potential causal factors in inducing osteoclast fusion^9,22,23^. However, there is no report of osteocytes influencing osteoclast fusion or other related mechanisms. In this study, live osteocytes played a more important role than osteoblasts in osteoclast fusion. We can hypothesize that such an impact probably relies most heavily on their membrane linked molecules. These findings open up a new horizon for understanding the key regulating cells for osteoclastogenesis. Previous studies have reported that MLO-Y4 osteocyte-like cells or osteocyte can regulate osteoclast formation and activation via M-CSF, OPG or RANKL expression and secretion^9,19,22–24^. Recently, an increasing evidence has surfaced indicating that osteocytes can not only secrete cytokines to affect other bone cells but can also potentially extend the process beyond the osteoblast layer to interact with bone marrow cells^25^. Based on our data, this suggests that molecules associated with the dendrites of osteocyte membranes possibly play an important role in the regulation of osteoclast fusion. Only further experiments can identify the exact osteocyte and osteoblast molecules involved in the process.

In addition, fused osteoclasts were sorted using a combination of this fluorescence technique with FACS. The results of this combination confirmed the concurrence of TG cells and G cells that had already been observed in previous experiments. But further studies are required to answer the question of why TG cells are present. Because TG cells contain both EGFP and tdTomato proteins, we propose two explanatory hypotheses. In the first place, it should be noted that the intracellular removal of proteins takes time^26^. At a very early stage after the fusion of a *Rosa*^*mTmG*^ BMM with a *Ctsk-Cre* BMM, the tdTomato protein is not entirely cleaned even though the tdTomato gene had already been excised previously. In the meantime, EGFP is translated^26^. Secondly, it is possible that fused GFP+ cells continue to fuse with red cells (tdTomato expressed cells). But mono-green cells seem to be morphologically larger and more mature. This suggests that they may be the cells that no longer fuse with red precursors. They may be involved in a later stage of the cells than TG cells (Fig. 3A–H). High-throughput RNA-seq also exposed the differences between these two populations with respect to gene expressions. Without further studies, it is impossible for us to determine the causes underlying the concurrence of TG cells and G cells. But this curious situation suggests possible heterogeneity with respect to the fusion of osteoclasts. TG cells exclusively expressed several important genes that are involved in mTOR signaling, a process which is generally believed to influence cell proliferation, apoptosis, and bone metabolism^27–34^. Consistently, KEGG analysis suggests that some of the genes in TG cells may participate in cell cycle processes and in apoptosis. Genes expressed by G cells, on the other hand, have potential roles in other processes, such as adhere junction, in starch and sucrose metabolism and in other glycan degradation respectively (S1). Hence, the distinctive profile of gene expression in TG cells potentially endows them with differential abilities in terms of proliferation, apoptosis, and metabolism. But these suggestions await further analysis to confirm.

## Conclusion

The fluorescence switch strategy which we have presented in this paper permits real-time visualization of osteoclast fusion. It also permits quantification of the dynamics of the entire fusion process. We are confident that this particular research tool, used in combination with other molecular bio-techniques, could contribute important insights into cellular processes in general and, more specifically, into the underlying mechanisms of osteoclast fusion.

## Materials and Methods

### Animals

*Cathepsin K-Cre* mice were provided by Dr. Yuuki Imai (Institute of Molecular and Cellular Biosciences, University of Tokyo, Japan)^13^. *Rosa*^*mTmG*^ mice were purchased from Jackson Lab (stock no: 007676, Bar Harbor, Maine, USA). This study was carried out in accordance with the guidelines of the State Key Laboratory of Oral Diseases and was approved by the Ethics Committee of the State Key Laboratory of Oral Diseases, Sichuan University.

### Cell culture materials

Cells were cultured with α-modified essential medium (α-MEM; Hyclone, South Logan, UT, USA), supplemented with 10% fetal bovine serum (FBS) and 1% penicillin-streptomycin (Gibco, Life Technologies, Carlsbad, CA, USA) in a 5% CO_2_ and 95% air incubator at 37 °C. Preosteoclast-like Raw264.7 cells and preosteoblast-like MC3T3-E1 cells were purchased from ATCC (ATCC, Manassas, VA, USA). Osteocyte-like MLO-Y4 cells were received as a gift from Prof. Lynda F. Bonewald^35^.

Fluorescent dyes DIL, Hoechst and DAPI were purchased from Thermo Fisher Scientific (Waltham, MA, USA). Recombinant mouse receptor activator of NF-ƙB ligand (RANKL), parathyroid hormone (PTH) and macrophage colony-stimulating factor (M-CSF) were obtained from Peprotech (Rocky Hill, NJ, USA). 1,25(OH)_2_D_3_ was acquired from BIOMOL Research Laboratories Inc. (Plymouth Meeting, PA, USA). Tartrate-resistant acid phosphatase reagents were purchased from Sigma (TRAP, Aldrich Sigma, St. Louis, MO, USA). RNA sample preparation was carried out according to the standard protocols of Invitrogen™ TRIzol™ (Thermo Fisher Scientific, Waltham, MA USA). Phosphate buffer saline (PBS), trypsin and 4% paraformaldehyde solution (4%PFA) were purchased from Boster China (Boster, Wuhan, China).

### Data Availability

All data generated or analyzed during this study are included in this published article and its Supplementary Information files.

## Cell Culture

### Co-culture systems

When Raw264.7 cells reached 80% confluence, the culture medium was discarded. The cells were then lightly washed twice with PBS (30 seconds per wash), then separately stained either with DIL (using a final concentration of 2uM) or with Hoechst (1ug/ml) in sterile PBS. Cells containing a DIL dying solution were incubated at 37 °C. After two minutes the solution was discarded. Meanwhile, cells which need to be labeled with Hoechst were then incubated with Hoechst solution at a temperature of 37 °C for 5 minutes. After removal of all dye solutions, the cells received a repeated PBS wash (3 times with 5 minutes for each wash). After washing, cells were detached with 0.25% trypsin for 1 minute and neutralized with a basic medium containing 10%FBS. The cells were then centrifuged at 200 g for 5 minutes and mixed together with a constitution ratio of 1:1. They were subsequently placed in a 96-well plate at a total density of 3*10^3^ cells. 24 hours later the medium was half-changed with 50 ng/ml M-CSF and 50 ng/ml RANKL. Medium with 50 ng/ml M-CSF and 50 ng/ml RANKL was subsequently half-changed every two days until the observations were completed.

Primary BMMs were isolated from the femur bone marrow of 2-month-old mice as previously described^36^. These BMMs from *Ctsk-Cre;Rosa*^*mTmG*^ mice were plated on an Osteo Assay Surface 96-well plate (Corning, Tewksbury, MA, USA), which was called collagen resorption analysis chamber in this manuscript, with 50 ng/ml M-CSF on the first day. Subsequently, on the third day, the entire medium was replaced with a fresh medium, to which 50 ng/ml M-CSF and 50 ng/ml RANKL were added. Afterward, a medium with 50 ng/ml M-CSF and 50 ng/ml RANKL was half-changed every two days. When the culture was complete, the cells were examined with a confocal microscope (Leica TCS SP5, Leica, Germany).

In the long-term tracking experiment, 50 ng/ml M-CSF was added on the first day following the plating of the BMMs. On the third day, BMMs from *Ctsk-Cre* and *Rosa*^*mTmG*^ mice were detached with 0.25% trypsin for 1 minute at 37 °C. Add complete culture medium with 10%FBS to inactive trypsin. The cell suspension was collected and centrifuged at 200 g for 5 minutes. The cells were then suspended and mixed at a 1:1 ratio. Later, they were seeded in culture dishes in a medium containing 50 ng/ml M-CSF and 50 ng/ml RANKL. The medium was half-changed every two days later.

Analysis concerning the effects of different concentrations of RANKL or PTH on osteoclast fusion used the same procedures of co-culturing BMMs from *Ctsk-Cre* and *Rosa*^*mTmG*^ mice. On the third day, we divided the co-cultured cells into 5 separate groups, each receiving a different concentration. The different concentrations of PTH or RANKL were as follows: 10 ng/ml, 25 ng/ml, 50 ng/ml, 75 ng/ml and 100 ng/ml. All received the same concentration of 50 ng/ml M-CSF. In PTH groups, only PTH and M-CSF were added without RANKL.

In our experiments on the impact of osteocytes and osteoblasts on osteoclast fusion, MC3T3-E1 or MLO-Y4 cells were cultured for 2 days in a 12-well plate to the confluence of 70–80% as the bottom layer. In order to detect the function of membrane molecules, the bottom cells were fixed with 4% PFA at room temperature for 10 minutes. They were then washed 5 times with PBS (5 minutes for each washing). This was defined as the “Dead” group. At that point 1:1-mixed BMMs from *Ctsk-Cre* and *Rosa*^*mTmG*^ mice were seeded upon the bottom layer. Meanwhile, to study the role of molecules secreted from the bottom layers, mixed BMMs were separated from live bottom cells using transwell chambers with 0.4 μm pore size (Corning, Tewksbury, MA, USA). This procedure permitted communication only between secreted molecules. This group was therefore named as the “Conditioned Medium (CM) group”. In the “Live” group the uppermost BMMs were directly plated on the live bottom cells. Twenty-four hours after the plating of the uppermost BMMs, all groups were treated with 10^−8^ M of 1, 25(OH)_2_D_3_. Half of the medium was replaced every 2 days. On the third, sixth and ninth day after treating with 1,25(OH)_2_D_3,_ images were produced using fluorescence microscopy (Olympus 1 × 71, Olympus, Tokyo, Japan). When the culture was complete, the fixed cells were stained for TRAP. These protocols were modified from what was reported in previous publications^24,37^.

## Histology

Two-month-old *Ctsk-Cre;Rosa*^*mTmG*^ mice were sacrificed and fixed overnight in 4%PFA at 4 °C. Fixed whole bodies were washed with PBS for one hour, followed by removal of the skins. These treated samples were examined using a stereo-fluorescence microscope with FITC and RFP channels (SteREO Discovery V20, Carl Zesis, Germany). After examination of the images produced by a stereo-microscopic imaging procedure, the femurs were immersed in a 30% sucrose solution overnight at 4 °C. Then samples were embedded in an OCT tissue-freezing medium (Leica, Germany) and placed upon dry ice to conduct non-decalcified frozen sectioning at 5 um thickness. The Kawamoto’s film method was used to attach the non-decalcified bone tissue on the slices^38^. The sections were later examined using a confocal microscope (Leica TCS SP5, Leica, Germany).

## Fluorescence Activated Cell Sorting (FACS) and High Throughput mRNA-sequencing (RNA-Seq)

Co-cultured *Ctsk-cre* and *Rosa*^*mTmG*^ BMMs were collected 8 days after treatment with 50 ng/ml M-CSF and 50 ng/ml RANKL. We sorted them used the FACS procedure to separate mono-GFP cells (defined as G cells) and double fluorescent cells which own both EGFP and tdTomato protein (defined as TG cells). The total RNA of these two populations of cells was then isolated and processed in order to conduct RNA sequencing. Subsequently, we performed a Kyoto Encyclopedia of Genes and Genomes analysis (KEGG analysis) as well as a gene ontology enrichment analysis (GO analysis) in accordance with published guidelines^39^.

## Statistical Analysis and Image Process Software

All experiments were repeated at least three times. The results of these procedures were then presented in the form of representative images. All error bars shown are the standard deviations (SD) calculated across triplicate experiments. Student t-tests or ANOVA with Tukey post-hoc tests were used, and the level of acceptable statistical significance was set at P < 0.05. All images were processed with Image Pro Plus 6.0 (Media Cybernetics, Rockville, MD, USA) and the FACS images were processed via FlowJo 10 (FlowJo LLC, USA).

## Electronic supplementary material

supplementary figures
