# Supplementary material for: Visual Osteoclast Fusion via A Fluorescence Method
Source: Sci Rep. 2018 Jul 5;8:10184. doi: 10.1038/s41598-018-28205-3 (PMC6033910; doi:10.1038/s41598-018-28205-3)
Supplement: Supplementary file 1 — supplementary figures [file 41598_2018_28205_MOESM1_ESM.pdf]

Supplementary Data

Visual Osteoclast Fusion via A Fluorescence Method

Boer Li[1,#], Fanyuan Yu[1,#], Fanzi Wu[1], Ke Wang[2], Feng Lou[1], Demao Zhang[1], Xueyang Liao[1], Bei Yin[1], Chenglin Wang[1], Ling Ye[1*]

1. State Key Laboratory of Oral Diseases, West China Hospital of Stomatology, Sichuan University, Chengdu, 610041, Sichuan, China
2. Department of Biomedical Sciences, Texas A&M College of Dentistry, Dallas, 75246, TX, USA
*Correspondence: yeling@scu.edu.cn
#These authors contributed equally to this work.

S1. The results of Kyoto Encyclopedia of Genes and Genomes (KEGG) analysis.

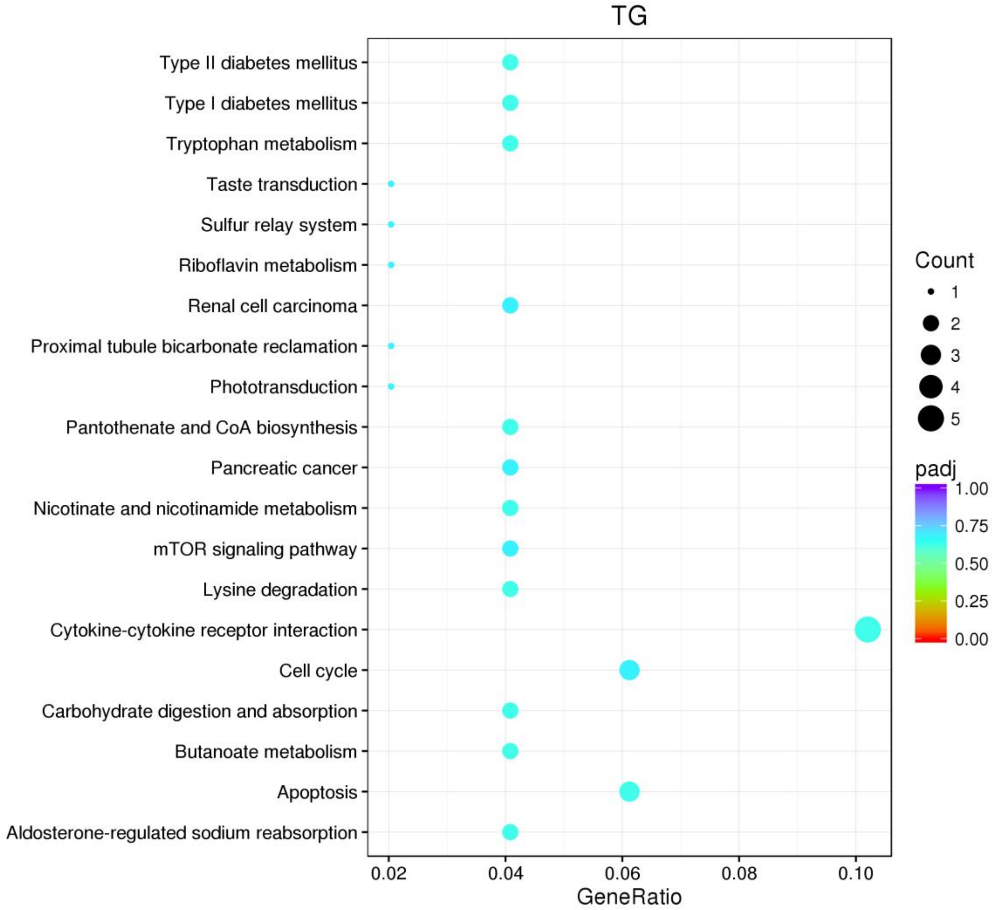

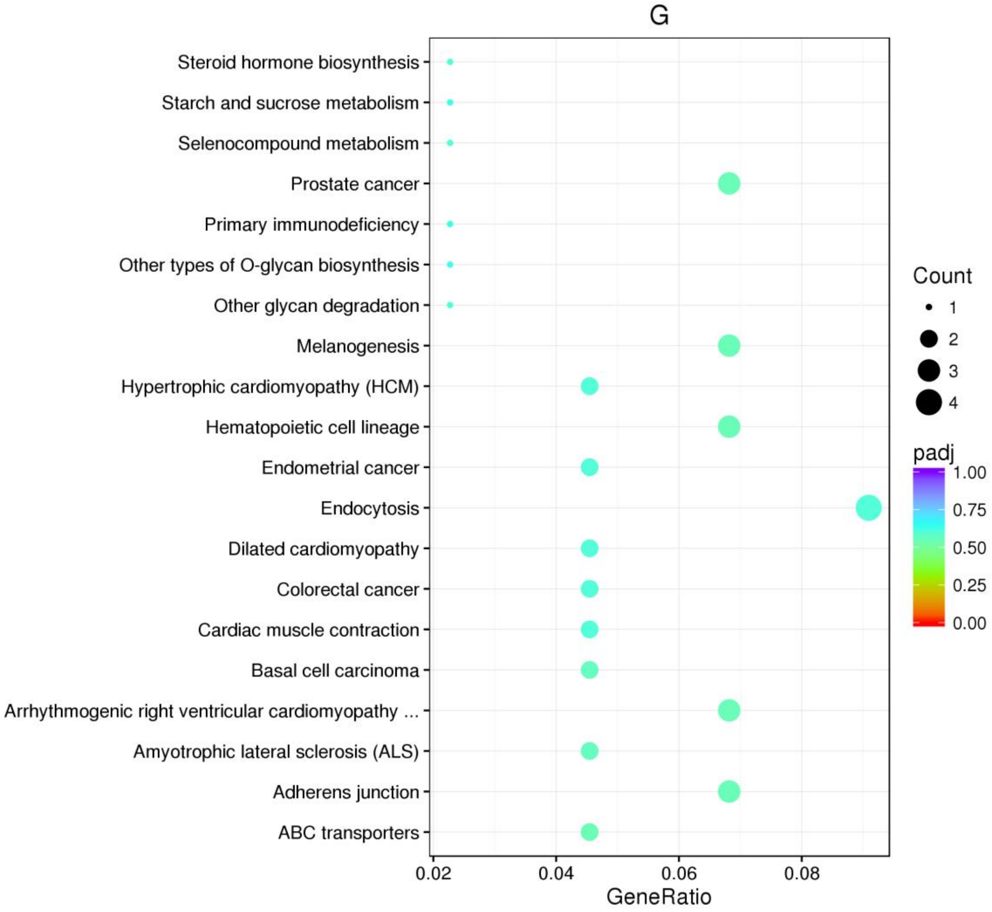

The KEGG analysis was conducted. Results indicate that the exclusive genes in TG cells and G cells play roles in different signaling pathways and cellular processes.

S2. The results of gene ontology enrichment analysis (GO analysis).

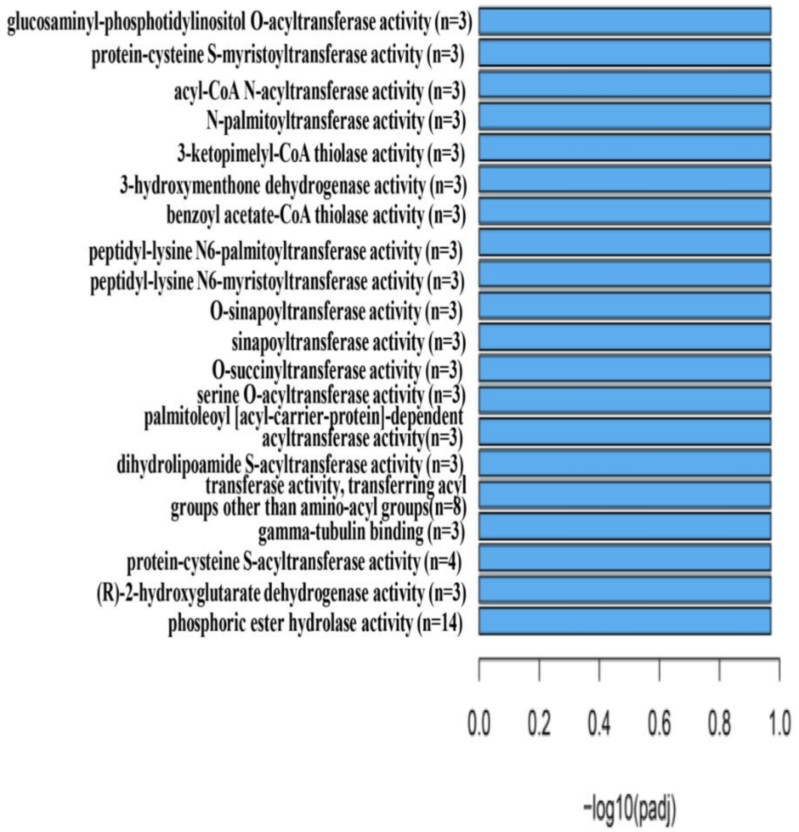

TG

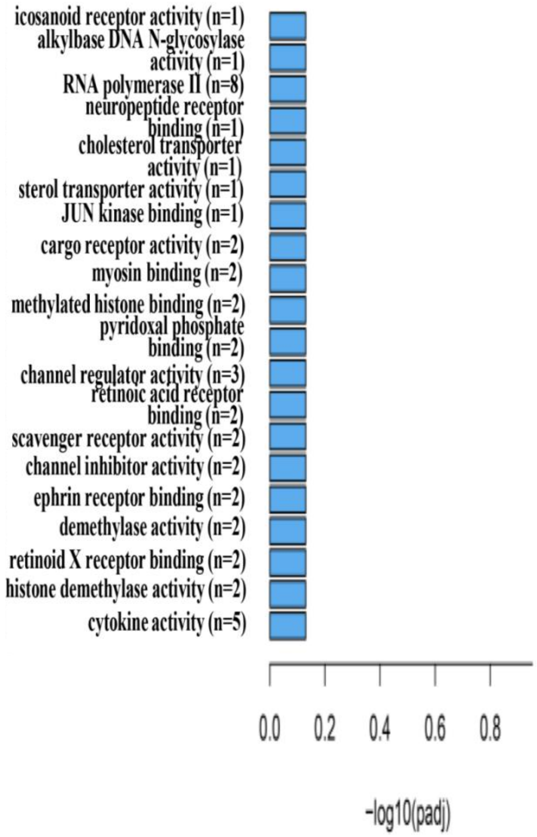

G

According to the results of gene ontology enrichment analysis (GO analysis), the exclusive genes in TG cells and G cells own different molecular functions (MF).

S3. The results of gene ontology enrichment analysis (GO analysis).

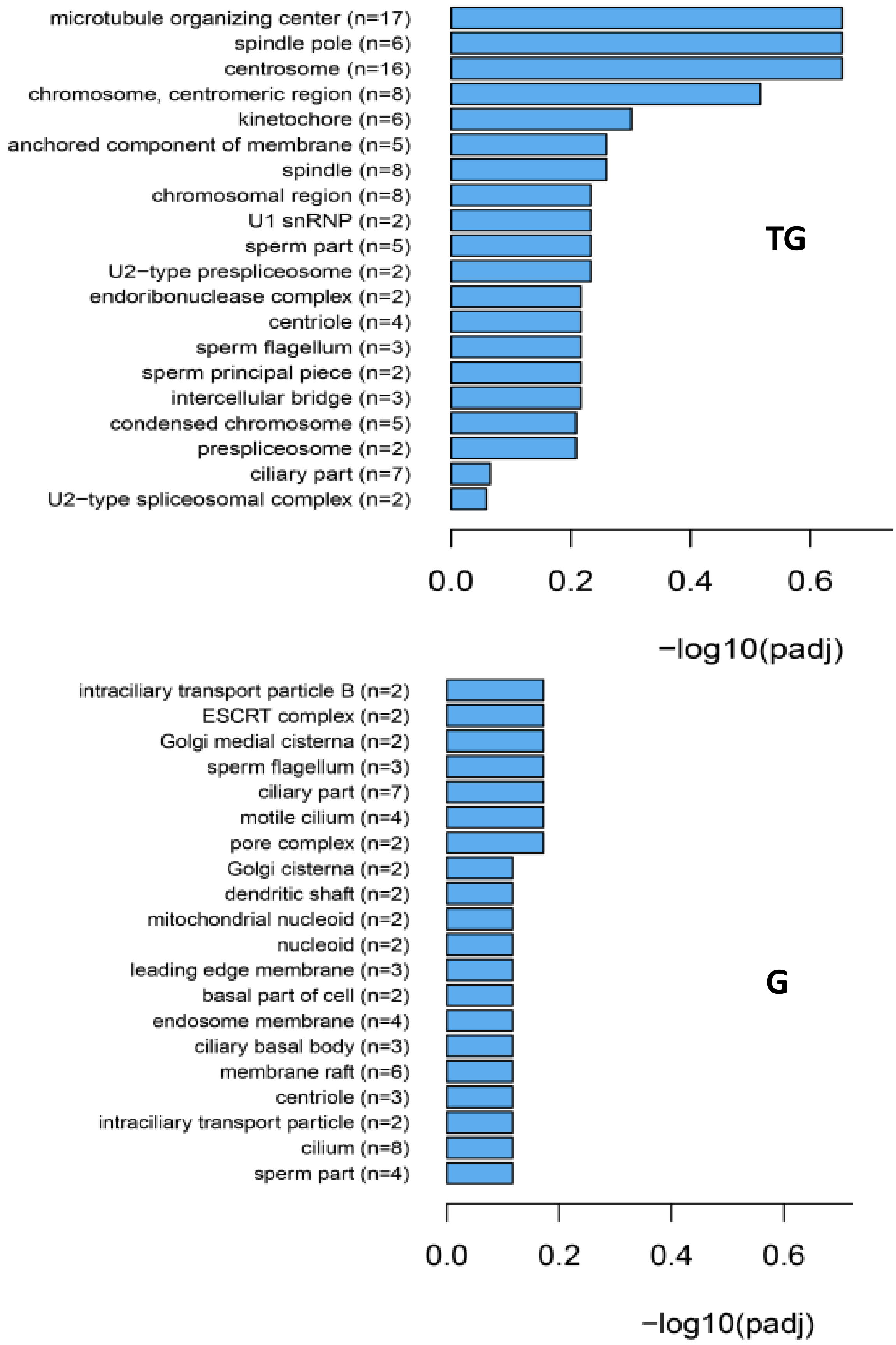

According to the results of gene ontology enrichment analysis (GO analysis), the exclusive genes in TG cells and G cells own different cellular components (CC).

S4. The results of gene ontology enrichment analysis (GO analysis).

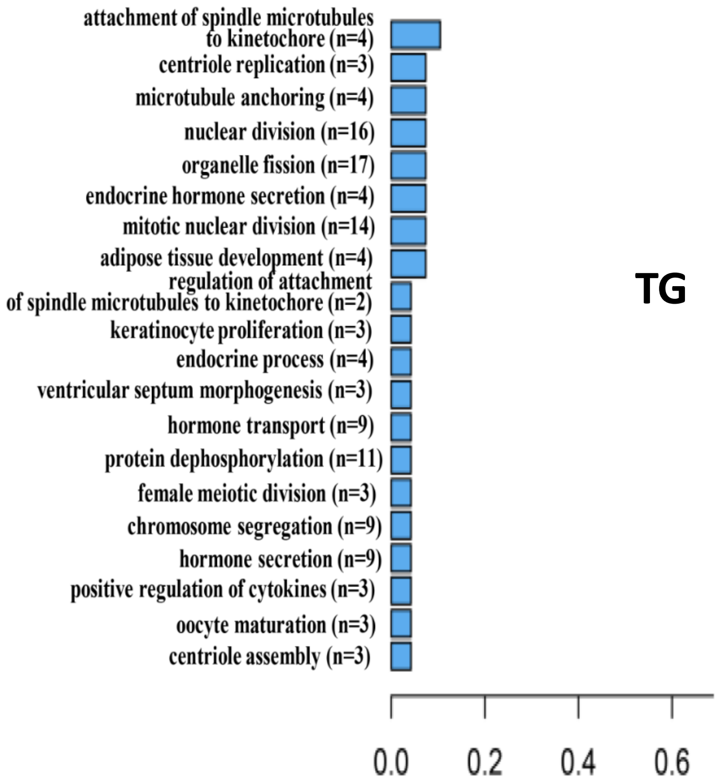

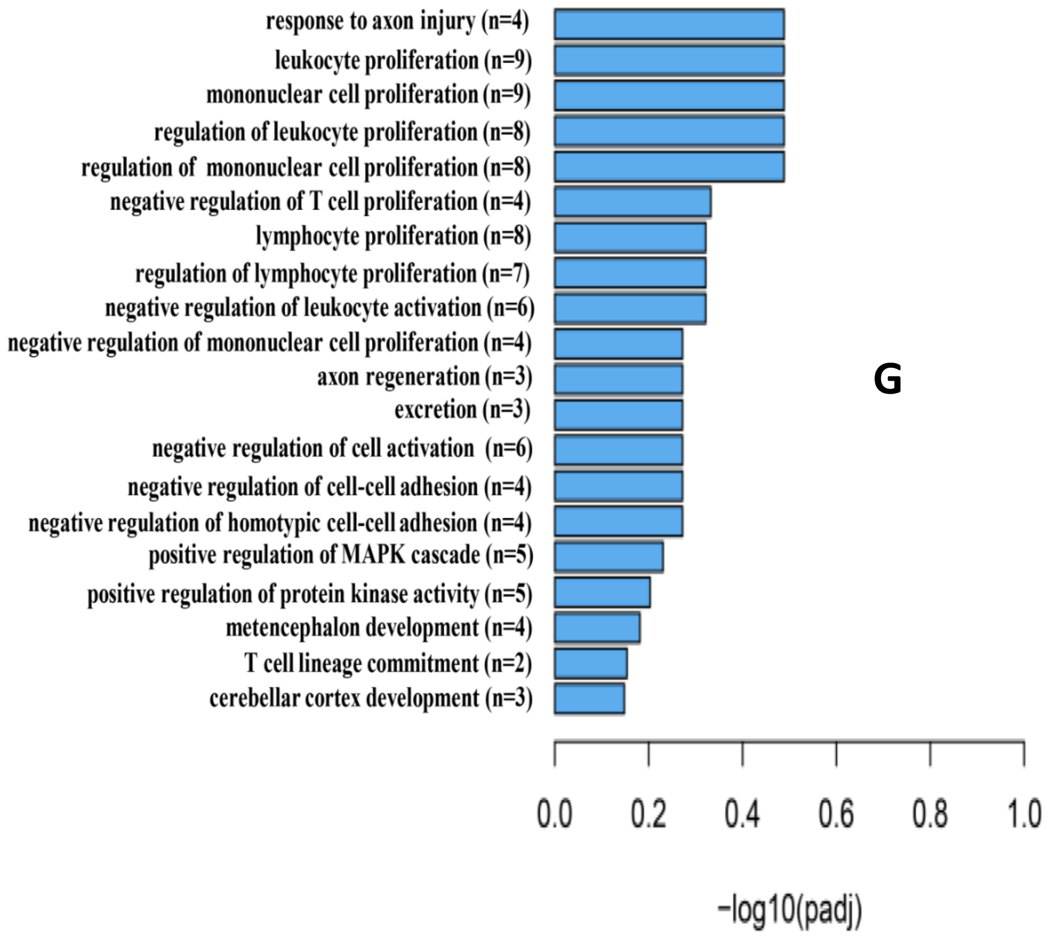

According to the results of gene ontology enrichment analysis (GO analysis), the exclusive genes in TG cells and G cells own different biological processes (BP).

S1-4: The results of Kyoto Encyclopedia of Genes and Genomes (KEGG) and gene ontology enrichment analysis (GO).

KEGG and GO enrichment analysis results indicated these exclusive genes in TG cells and G cells participated in different signaling pathways and events. Also, they were predicted to perform functions in different biological processes(BP), molecular functions(MF) and cellular components(CC).

S5. Fluorescent image of the femur from *Ctsk-Cre; Rosa^{mTmG}* mice

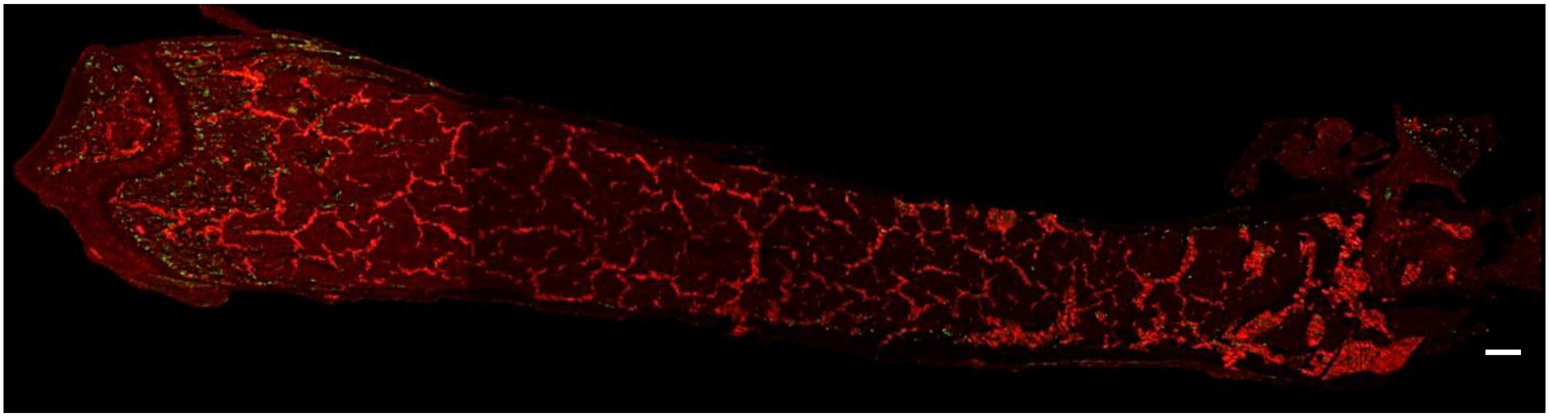

The GFP+ cells were located principally in trabecular bone and cortical bone surfaces whose densities were dramatically higher in epiphysis. Scale bar: 100um

S6. The distal end of the femur from *Ctsk-Cre; Rosa^{mTmG}* mice

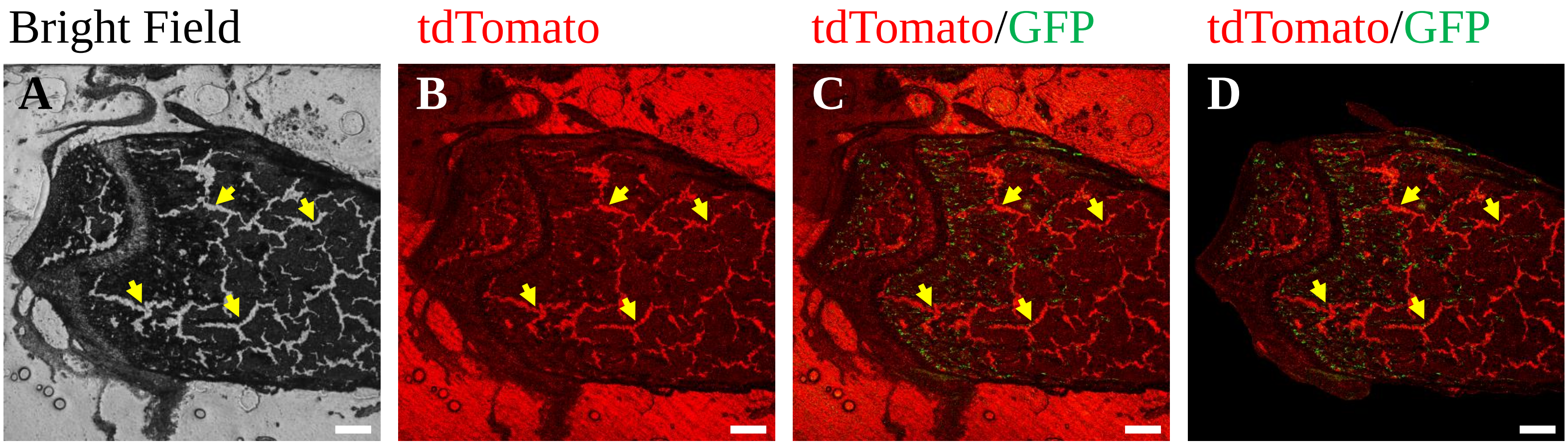

A: The bright field of the frozen section, the yellow arrows indicated the cracks of the bone marrow. B: The tdTomato fluorescence of the femur, the yellow arrows indicated the same cracks as in panel A. C: The merged image of tdTomato and green fluorescence of the femur. D: The merged image showed in area of interest. The area of interest was selected when the image was captured by the microscope, the yellow arrows indicated the same cracks as in panel A. Scale bar: 100μm.

The frozen section of the non-decalcified bone tissue is the reason of these cracks in the bone marrow. The transfer films we used to attach the non-decalcified bone tissue on the slice are responsible for the autofluorescence in the Figure2B and S6. Due to the technical limitation, we could not avoid the autofluorescence by the transfer films in the bone marrow.

S7. Fluorescent image of mixed BMMs from *Ctsk-Cre* and *Rosa^{mTmG}* mice cultured without RANKL

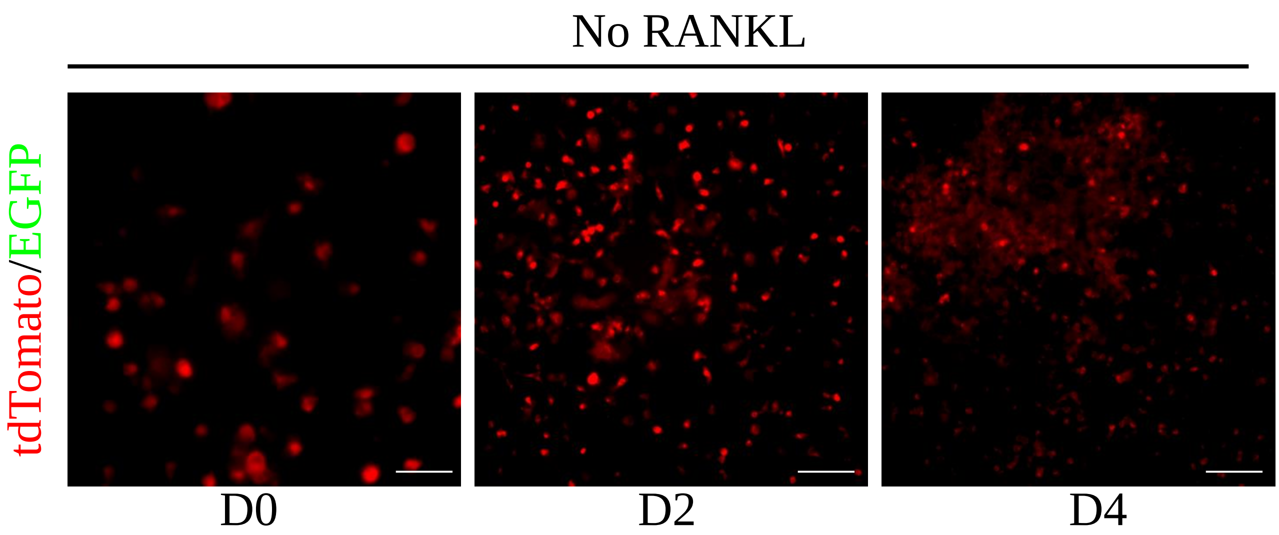

The Fluorescent detection showed that BMM did not fuse and became GFP+ cells without RANKL induction. The cells were the 1:1-mixed BMMs harvested from Ctsk-Cre and Rosa^{mTmG} mice and cultured without RANKL for 0day, 2days and 4 days.

S8. Flow cytometry analysis of TRAP expression for GFP+ cells

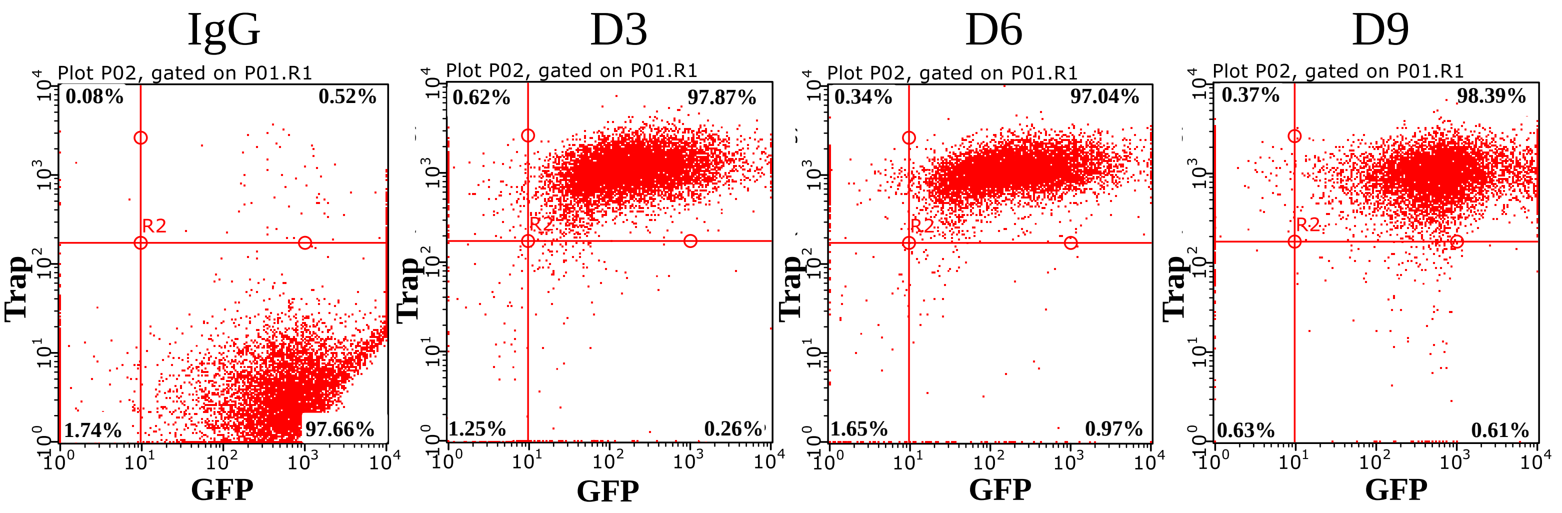

Flow cytometry analysis was performed using Staining Kit (#ebioscience 00-5523) in accordance with its protocol. It showed that almost all GFP+ cells were TRAP positive.
